# Patterns of smartphone dependence and predictive factors in Chinese college freshmen: a latent profile analysis

**DOI:** 10.3389/fpsyg.2025.1592875

**Published:** 2025-06-18

**Authors:** Liang Chen, Zhanyang Yuan, Minhui Jiang

**Affiliations:** ^1^Mental Health Education Center, Zhejiang A&F University, Hangzhou, China; ^2^Mental Health Education Center, Xinyang Normal University, Xinyang, China; ^3^Department of Psychology, Wuxi Maternal and Child Health Hospital, Wuxi, China

**Keywords:** college freshmen, smartphone dependence, psychological resilience, latent profile analysis (LPA), predictive factors

## Abstract

**Objective:**

To explore the latent classes of smartphone dependency among college freshmen and analyze the predictive factors of these classes, to provide a basis for the development of targeted intervention strategies.

**Methods:**

A total of 4,863 college freshmen from Zhejiang A&F University in 2023 were selected for the questionnaire survey by using the Socio-demographic information, Smartphone Dependency Scale and Psychological Resilience Scale, followed by latent profile analysis and logistic regression for data analysis.

**Results:**

Smartphone dependency among college freshmen was categorized into four classes: normal use group (11.2%), mild dependency group (37.4%), moderate dependency group (41.3%), and high dependency g**r**oup (10.1%). Negative life events, suicidal ideation, and psychological resilience were significantly associated with the latent classes of smartphone dependency among college freshmen.

**Conclusion:**

There is heterogeneity in smartphone dependency among college freshmen, those experiencing suicidal ideation exhibit increased sensitivity to smartphone dependency, those encountering negative life events are more prone to smartphone dependency, while psychological resilience acts as a protective factor against smartphone dependency among college freshmen. These insights not only enhance our understanding of the characteristics of smartphone dependency among college freshmen, but also provide a scientific basis for psychological health education and personalized interventions.

## 1 Introduction

With the continuous progress of 5G technology and smart phone technology, smartphones have penetrated into all aspects of our daily life. According to the data of China Internet Network Information Center, as of May 2024, the number of mobile Internet users in China has reached 1.091 billion. Although smartphones have become important tools for work, communication, entertainment, transactions, and shopping, excessive reliance on smartphones has become a critical public health issue, negatively impacting people’s mental health and social behavior, especially among college students (Park and Lee, 2012). A recent meta-analysis study indicates that over the past decade, the prevalence of smartphone addiction among Chinese college students has shown a continuously rising trend (Lyu et al., 2024). The prevalence of smartphone addiction among Chinese university students typically exceeds 20% (Long et al., 2016; Mei et al., 2023). Notably, previous research has shown that college freshmen face a nearly 40% higher risk of smartphone addiction (Liu et al., 2022). Empirical research have shown that excessive smartphone dependence can have a direct or indirect impact on learning (Sunday et al., 2021), emotions, behaviors and self-growth (Felisoni and Godoi, 2018), which, more seriously, may lead to interpersonal problems (Kyoung et al., 2017), depression (Demirci et al., 2015; Geng et al., 2021), anxiety (Demirci et al., 2015; Geng et al., 2021), sleep problems (Abuhamdah and Naser, 2023; Demirci et al., 2015; Hamvai et al., 2023), and even the risk of non-suicidal self-injury increases (Long et al., 2024). Therefore, identifying the patterns of smartphone dependence among college freshmen and the associated risks and protective factors is of practical significance. This information can guide prevention and intervention efforts to promote the rational use of smartphones.

Existing research predominantly adopts variable-centered approaches to assess smartphones addiction, which is often bifurcated into “addicted” and “non-addicted” categories (Elhai et al., 2020), failing to capture the heterogeneous characteristics of smartphone addiction behaviors and provide more targeted prevention and intervention for different patterns. Therefore, LPA, as a person-centered approach, can classify individuals and identify group heterogeneity based on their distinct response patterns to continuously scored test items. LPA is used in many fields such as psychology and psychiatry, and its classification accuracy and effectiveness are significantly higher than traditional classification (Notelaers et al., 2006). Previous research has indicated that individuals exhibit varying mechanisms underlying mobile phone addiction, leading to the emergence of distinct types of this behavior (Elhai et al., 2019; Kim et al., 2016; Lv et al., 2023). In several studies conducted in China (Li et al., 2024; Lv et al., 2023; Yue et al., 2021), three potential classes of college students’ smartphone addiction were identified: the low-risk group, the moderate-risk group, and the high-risk group. A study conducted with 300 undergraduate students in the United States revealed three different potential categories (Elhai et al., 2019): a mild category (30.7%), a moderate category (48.4%), and a severe category (20.9%). However, other studies do not support these findings. Elhai et al. (2020) found two subgroups of smartphone addiction patterns among 286 American college students: “the group with no problems in smartphone use” and “the group with problematic smartphone use.” Another study involving 1,123 undergraduate students in China identified four distinct potential categories of smartphone addiction (Hong et al., 2022): the low-risk group (11.0%), the non-avoidant moderate-risk group (24.1%), the avoidant moderate-risk group (35.5%), and the high-risk group (29.4%). Although the aforementioned studies utilized LPA to identify subgroups of individuals with smartphone addiction, there still exist significant discrepancies among these research due to differences in cultural difference, research samples and the methods of measuring mobile phone addiction. Firstly, these studies have shown varying results in the number of profiles and the characteristics of each profile. Secondly, previous research has mainly focused on college students and has not fully reflected the underlying patterns of smartphone addiction among college freshmen. This study will employ LPA to thoroughly explore the latent classes of smartphone addiction among college freshmen, providing a more precise basis for subsequent interventions.

Numerous previous studies have investigated the risk and protective factors associated with smartphone addiction. At the individual level, multiple studies have demonstrated that adverse psychological states such as social anxiety (Yilmaz et al., 2024), loneliness (Shi et al., 2023), depression (Shi et al., 2023; Yilmaz et al., 2024) serve as risk factors for addiction, while psychological resilience, defined as the ability to adaptively face adversity, trauma, tragedy and threats through emotional regulation, self-efficacy, and cognitive flexibility (Connor and Davidson, 2003; Rutter, 1987), has received widespread attention as a potential buffer against behavioral addictions. Cross-sectional studies consistently reported inverse correlations between resilience and smartphone dependency, wherein resilient individuals demonstrate enhanced self-control and reduced escapism-driven device usage (Robertson et al., 2018; Wu et al., 2024). Another study also have shown that individuals’ perceived social support and resilience were significant predictors for mobile media addiction (Bilgin and Taş, 2018). Furthermore, recent research has revealed that enhancing psychological resilience and life satisfaction may effectively attenuate the development of mobile phone short video dependence among individuals with childhood adverse experiences (Xue et al., 2025).

The ecosystem theory (Bronfenbrenner, 1986) emphasizes that individual development is intrinsically linked to the surrounding environment. The social ecological model (Golden et al., 2015) further elaborates that an individual’s social behavior is not merely a function of personal choices or intentions, but is also profoundly shaped by the broader social and physical environment in which they are embedded. So, smartphone dependency is produced by the dynamic interaction between individual traits and the environmental system. At the microsystem level, familial dysfunction (e.g., parental neglect) and peer conflict amplify the risk of mobile phone dependence through difficulties in emotional regulation (Gao et al., 2022; Lian et al., 2023). Conversely, macrosystem factors such as socioeconomic status, further modulate patterns of mobile phone use (Xiong et al., 2023). An empirical study involving 1,399 Chinese adolescents demonstrated a significant positive correlation between negative life events and smartphone addiction (Ji et al., 2025). A longitudinal study of undergraduate students also showed that baseline stressful life events were significantly associated with problematic smartphone use 1 year later (Zhao et al., 2021). Notably, emerging evidence highlights the mediating role of negative life events (e.g., academic failure, interpersonal discord) in exacerbating smartphone dependency, particularly among students with preexisting vulnerabilities such as suicidal ideation (Huang et al., 2022; Yang et al., 2021). Moreover, multiple studies have indicated a correlation between excessive smartphone use and adolescent depression as well as suicidal behaviors (Kim et al., 2019; Shinetsetseg et al., 2022). Another study involving a convenience sample of 1,042 Chinese college students indicated a positive correlation between mobile phone addiction and suicidal ideation induced by depression (Hu et al., 2022). However, how these factors contribute to different phenotypes of mobile phone dependence remains to be further explored. Our study integrates individual characteristics, microsystems, and macrosystems to provide a more comprehensive explanation of smartphone addiction mechanisms.

Therefore, this study employs LPA to delineate the subtypes of smartphone dependency among Chinese college freshmen—a population undergoing critical transitions in autonomy and psychosocial adaptation, and explores predictors of latent classes of smartphone dependence based on ecological systems theory. This study aims to advance the theoretical and practical understanding of smartphone dependence, providing insights that can inform interventions and policies aimed at promoting healthy smartphone use.

## 2 Materials and methods

### 2.1 Participants

The data of this study comes from the psychological survey of freshmen in a university in Hangzhou in 2023, with a total of 4,863 students, detailed demographic statistics of the sample can be seen in Supplementary Table S1. The psychological survey was conducted by the University Student Mental Health Education Center within 1 month of the students’ enrollment. The students were organized by their respective colleges to take the unified online test in the classroom. The study received approval from the Ethics Committee of Zhejiang A&F University (no. ZAFU2023/0902-1).

### 2.2 Methods

#### 2.2.1 Predictive factors

According to the ecosystem theory, we categorized the predictive factors influencing the heterogeneity of mobile phone addiction among college freshmen into: individual characteristics including gender (female, male), negative life events (yes, no), left-behind experience (yes, no), mental illness (yes, no), suicidal ideation (yes, no) and psychological resilience; microsystems variables including family ranking (only child, non-only child), family structure (complete, single parent) and peer relationship (harmonious, discordant); macrosystems variables including origin (urban, rural) and family economic status (well-off, common, poor). The single-item screening method was used to measure negative life events and suicidal ideation, such as “Have you experienced negative life events such as academic setbacks, interpersonal conflicts, or family adversities in the past year?” and “Have you had suicidal thoughts in the past year?” Single-item measures have been demonstrated to be reliably and effectively applied in large-scale studies (Matthews et al., 2022).

#### 2.2.2 Mobile phone addiction index scale

The Mobile Phone Addiction Index (MPAI), originally developed by Leung (2008) and revised by Huang et al. (2014), consists of 17 items, including four dimensions: loss of control (7 items, e.g., “You have attempted to spend less time on your mobile phone but are unable to”), withdrawal (4 items, e.g., “You find it difficult to turn your phone off.”), escapism (3 items, e.g., “I use my phone to forget about my problems”) and inefficiency (3 items, e.g., “The time spent on your phone directly reduces our work efficiency”). The scale retains the original structure but modifies items for cultural relevance. Participants rated each item on a 5-point scale from 1 (almost never) to 5 (almost). The higher the total score on the scale, the higher the level of smartphone addiction. Mobile phone addiction is classified by severity, with a total score of 34–51 indicating mild addiction, 51–68 indicating moderate addiction, and 68–85 indicating severe addiction. These thresholds have been widely applied in Chinese student populations (Li et al., 2024). In this study, the total scale’s Cronbach’s *α* coefficient was 0.92, with α coefficients for each dimension (loss of control, withdrawal, escapism, inefficiency) ranging between 0.85–0.91, indicating good internal consistency of the scale.

#### 2.2.3 Psychological resilience questionnaire

Psychological Resilience Questionnaire, originally developed by Connor and Davidson (2003) and revised by Yu and Zhang (2007), comprises of 25 items, including three dimensions: resilience (13 items, e.g., “When things look hopeless, I do not give up”), strength (8 items, e.g., “I am able to adapt to change”) and optimism (4 items, e.g., “I Can deal with whatever comes”). Participants rated each item on a 5-point scale from 0 (never) to 4 (always). The higher the total score of the scale, the stronger the individual’s psychological resilience. In this study, the total scale’s Cronbach’s α coefficient was 0.96, with α coefficients for all dimensions (resilience, strength, optimism) exceeding 0.90, indicating extremely high reliability.

#### 2.2.4 Data analysis

With unified guidance, group testing was conducted on a class basis, and questionnaires were collected uniformly. SPSS 26.0 was employed for descriptive statistics, univariate analysis and logistic regression analysis. Mplus 8.0 was used to conduct latent profile analysis on four dimensions of mobile phone dependence. Models with 1–5 latent classes were examined, and missing data were addressed with maximum likelihood estimation. To prevent convergence on solutions at a local maximum, we conducted the LPA with 1,000 random starting values and 300 final-stage optimizations. Model selection was based on statistical evaluation indicators and theoretical considerations. The main evaluation indicators include Akaike Information Criterion (AIC) and Bayesian Information Criterion (BIC), adjusted Bayesian information criterion (aBIC), Entropy index (Entropy), Lo-Mendel-Rubin likelihood ratio test (LMRT) and Bootstrap likelihood ratio test (BLRT) (Vermunt and Magidson, 2003). Wherein, the smaller the statistics of AIC, BIC and aBIC, the better the fitting effect of the model. The closer the Entropy value is to 1, the higher the classification accuracy of the model. The *p* value corresponding to LMRT and BLRT reached a significant level, indicating that the K-class model is better than the k-1 class model (Nylund et al., 2008). The best model was selected according to the principles of model interpretability and simplicity. After the LPA confirms the latent classes, we conducted univariate analysis, comparing categorical predictor variables through chi-square tests and continuous predictor variables through analysis of variance to assess the impact of each predictor on latent classes. All predictors that were significant in the univariate analysis were incorporated into the logistic regression model to explore the predictive factors of subgroups of smartphone dependence.

## 3 Results

### 3.1 Test for common method bias

The main data for this study all come from a questionnaire survey, and there may be common method biases. Therefore, when designing the survey questionnaire, some methods were taken to control the mutual influence between scales (Kock et al., 2021). Anonymous surveys and reverse scoring questions were used in the study of test common method bias using Harman’s single-factor test (Podsakoff et al., 2003). The results show that the amount of variation explained by the first factor is 27.66% (less than the critical value of 40%), indicating that this study does not have a serious common method bias.

### 3.2 Latent profile analysis of the smartphone dependence among college freshmen

**Figure 1:**
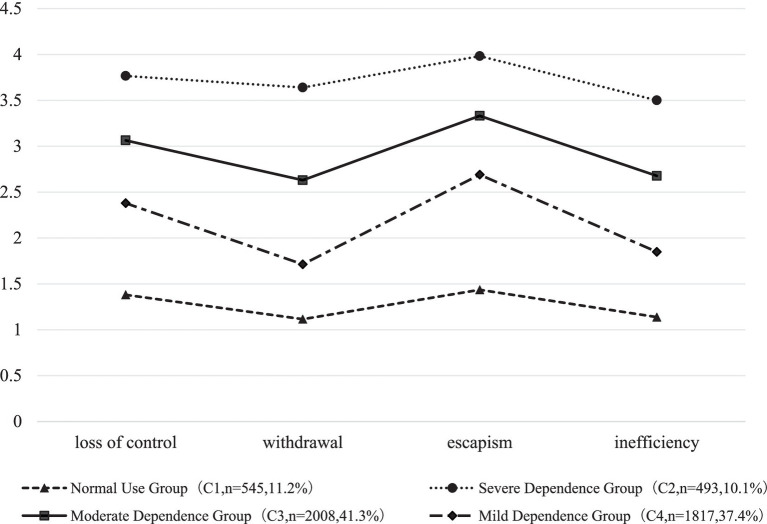
The average scores of the four latent classes of the smartphone dependence among college freshmen in the four dimensions of MPAI.

In this study, 1–5 latent class models were selected respectively, with results showing that (Table 1), the values of AIC, BIC and aBIC all decreased as the number of latent classes model increased, until the 4-class model decreased obviously, and the Entropy values of types 2, 3, 4 and 5 all exceeded 0.8, with the highest entropy value observed for the 4-class model, reflecting that the 4-class model had the best class accuracy. In addition, the LMRT and BLRT values of the 4-class model were all statistically significant (*p* < 0.001). Meanwhile, according to the recommendation by Nylund et al. (2008), when increasing the number of classes only brings minor improvements in AIC, BIC, and aBIC values without a significant enhancement in Entropy, a more parsimonious model should be prioritized. Moreover, the newly added fifth category in the 5-class model exhibits high overlap in dependency dimension scores with the severe dependency group in the 4-class model, lacking distinctive behavioral or psychological characteristics. Therefore, considering comprehensively, the 4-class model was selected as the best fitting models. The matrix of the attribution probability of this model is shown in Table 2, indicating that the average probability of college freshmen belonging to each class ranges from 94 to 96%, which indicates that the model results of the four classes are credible. On this basis, the answer probability diagram of four latent classes in the four dimensions of smartphone dependence was further obtained (Figure 1).

**Table 1:** Latent profile analysis results.

Model	AIC	BIC	aBIC	Entropy	LMRT	BLRT	Class probability (%)				
							C1	C2	C3	C4	C5
1	141633.98	141697.87	141666.09	–	–	–					
2	131968.09	132071.91	132021.07	0.86	<0.001	<0.001	44.66	55.34			
3	127853.65	127996.41	127926.51	0.83	<0.001	<0.001	13.32	45.61	41.07		
4	124501.54	124683.25	124594.27	0.91	<0.001	<0.001	11.21	10.14	41.29	37.36	
5	123085.80	123306.44	123306.40	0.88	<0.001	<0.001	8.55	21.29	28.02	33.50	8.67

AIC, Akaike information criteria; BIC, Bayesian information criteria; aBIC, adjusted Bayesian information criterion; BLRT, Bootstrap likelihood ratio test; VLMR, Vuong-Lo–Mendell–Rubin test.

**Table 2:** Average attribution probability (%) of participants in each latent class.

Class	C1	C2	C3	C4
C1	0.96	0.00	0.04	0.00
C2	0.00	0.95	0.03	0.02
C3	0.02	0.04	0.94	0.00
C4	0.00	0.07	0.00	0.94

Class 1 (C1), Normal Use Group; Class 2 (C2), Severe Dependence Group; Class 3, (C3) Moderate Dependence Group; Class 4 (C4), Mild Dependence Group.

As shown in Figure 1, college freshmen show no intersection in the four dimensions of loss of control, withdrawal, escapism, and inefficiency regarding smartphone dependency, and the forms of the four classes tend to be consistent. The mean values of the dimensions of the 1-class range from 1.11 to 1.44, and the scores of each dimension are lower than those of other classes, with a total of 545 individuals, accounting for 11.2%, named as “Normal Use Group,” the mean values of dimensions of the 2-class range from 3.50 to 3.98, and the scores of each dimension are higher than those of other classes, with a total of 493 individuals, accounting for 10.1%, named as “Severe Dependence Group,” the mean values of dimensions in 3-class range from 2.33 to 3.06, with a total of 2008 individuals, accounting for 41.3%, while the 4-class has mean values of a dimension of 1.71–2.69, with a total of 1817 individuals, accounting for 37.4%. Therefore, the 3-class and the 4-class were named as “Moderate Dependence Group” and “Mild Dependence Group” respectively. Among the three groups of Mild, Moderate, and Severe Dependence Groups, the mean score for the escapism dimension is the highest in all.

### 3.3 Predictors of latent classes of the smartphone dependence among college freshmen

Univariate analysis of different latent classes of the smartphone dependence among college freshmen shows that there are significant differences in gender, family economic status, peer relationship, negative life events, left-behind experience, suicidal ideation and mental resilience among different classes of participants, but there are no significant differences in origin, family ranking, family structure and mental illness (Table 3). The results of multinomial logistic regression of different latent classes of the smartphone dependence among college freshmen show that negative life events, suicidal ideation and psychological resilience affect the latent classes of the smartphone dependence among college freshmen to varying degrees (Table 4). Specifically, compared with the normal use group, the higher the total score of resilience, the lower the risk of college freshmen being classified as mild, moderate and severe smartphone dependence groups by 4, 6 and 7%, respectively. Compared with the mild dependence group, the higher the total score of resilience, the lower the risk of college freshmen being classified as moderate and severe smartphone dependence groups by 3%. Compared with the normal use group, college freshmen with suicidal ideation are 1.84 times and 2.61 times more likely to belong to the moderate and severe smartphone dependence group than those without suicidal ideation. Compared with the mild dependence group, college freshmen with suicidal ideation are 1.40 times and 1.98 times more likely to belong to the moderate and severe smartphone dependence group, and 1.41 times more likely to belong to the severe smartphone dependence group. In addition, compared with the light dependence group, the probability that college freshmen with negative life events belong to the heavy cell phone dependence group is 1.40 times higher than that without negative life events.

**Table 3:** Univariate analysis of social-demographic factors and psychological resilience factors and latent classes of the smartphone dependence among college freshmen.

Variable	Classification	C1	C2	C3	C4	*χ*^2^/F
Gender (*n*, %)	Female	251 (46.1)	267 (54.2)	1,063 (52.9)	933 (51.3)	9.48^*^
	Male	294 (53.9)	226 (45.8)	945 (47.1)	884 (48.7)	
Origin (*n*, %)	Urban	186 (34.1)	149 (30.2)	683 (34.0)	623 (34.3)	3.08
	Rural	359 (65.9)	344 (69.8)	1,325 (66.0)	1,194 (65.7)	
Family ranking (*n*, %)	Only child	206 (37.8)	181 (36.7)	702 (35.0)	691 (38.0)	4.27
	Non-only child	339 (62.2)	312 (63.3)	1,306 (65.0)	1,126 (62.0)	
Family structure (*n*, %)	Complete	481 (88.3)	429 (87.0)	1803 (89.8)	1,643 (90.4)	5.97
	Single parent	64 (11.7)	64 (13.0)	205 (10.2)	174 (9.6)	
Family economic status (*n*, %)	Well-off	142 (26.1)	89 (18.1)	411 (20.5)	403 (22.2)	18.19^**^
	Common	362 (66.4)	350 (71.0)	1,420 (70.7)	1,282 (70.6)	
	Poor	41 (7.5)	54 (11.0)	177 (8.8)	132 (7.3)	
Peer relationship (*n*, %)	Harmonious	519 (95.2)	441 (89.5)	1860 (92.6)	1712 (94.2)	18.47^***^
	Discordant	26 (4.8)	52 (10.5)	148 (7.4)	105 (5.8)	
Negative life events (*n*, %)	Yes	31 (5.7)	66 (13.4)	211 (10.5)	131 (7.2)	31.48^***^
	No	514 (94.3)	427 (86.6)	1797 (89.5)	1,686 (92.8)	
Left-behind experience (*n*, %)	Yes	71 (13.0)	109 (22.1)	355 (17.7)	264 (14.5)	23.24^***^
	No	474 (87.0)	384 (77.9)	1,653 (82.3)	1,553 (85.5)	
Mental illness (*n*, %)	Yes	8 (1.5)	12 (2.4)	44 (2.2)	29 (1.6)	3.09
	No	537 (98.5)	481 (97.6)	1,964 (97.8)	1,788 (98.4)	
Suicidal ideation (*n*, %)	Yes	27 (5.0)	109 (22.1)	300 (14.9)	160 (8.8)	105.10^***^
	No	518 (95.0)	384 (77.9)	1,708 (85.1)	1,657 (91.2)	
Psychological resilience (Mean ± SD)	Resilience	53.23 ± 11.33	41.69 ± 9.30	42.97 ± 8.31	46.59 ± 8.57	230.21^***^
	Strength	33.77 ± 6.89	27.23 ± 5.41	28.03 ± 4.92	30.49 ± 5.00	224.40^***^
	Optimism	15.91 ± 3.67	13.49 ± 2.79	13.59 ± 2.66	14.41 ± 2.72	110.53^***^
	RISC total score	102.91 ± 21.01	82.41 ± 16.36	84.60 ± 14.88	91.49 ± 15.19	232.04^***^

^*^p < 0.05, ^**^p < 0.01, ^***^p < 0.001; RISC, Resilience scale.

**Table 4:** Multinomial logistic regression analysis of latent classes of the smartphone dependence among college freshmen (odds ratio and 95% confidence interval).

Predictor variable	C2 vs. C1	C3 vs. C1	C4 vs. C1	C2 vs. C4	C3vs. C4	C2 vs. C3
Gender (female vs. male)	1.20 (0.93–1.55)	1.16 (0.95–1.43)	1.14 (0.93–1.39)	1.06 (0.86–1.30)	1.02 (0.90–1.17)	1.03 (0.85–1.26)
Family economic status (poverty as the reference group)						
Well-off	0.97 (0.57–1.64)	1.13 (0.74–1.74)	1.16 (0.76–1.77)	0.84 (0.55–1.27)	0.98 (0.74–1.29)	0.85 (0.57–1.27)
Common	1.00 (0.63–1.60)	1.09 (0.74–1.61)	1.18 (0.80–1.74)	0.85 (0.60–1.21)	0.93 (0.72–1.19)	0.92 (0.66–1.29)
Peer relationship (harmony vs. disharmony)	0.80 (0.47–1.37)	0.97 (0.60–1.54)	0.97 (0.61–1.54)	0.83 (0.57–1.20)	1.00 (0.76–1.31)	0.83 (0.58–1.18)
Negative life events (yes vs. no)	1.40 (0.87–2.25)	1.25 (0.82–1.89)	0.99 (0.65–1.51)	1.40 (1.01–1.96)^*^	1.25 (0.99–1.59)	1.12 (0.82–1.52)
Left-behind experience (yes vs. no)	1.19 (0.84–1.70)	1.02 (0.76–1.38)	0.94 (0.70–1.26)	1.28 (0.98–1.66)	1.09 (0.91–1.31)	1.17 (0.91–1.51)
Suicidal ideation (yes vs. no)	2.61 (1.62–4.19)^***^	1.84 (1.20–2.85)^**^	1.31 (0.85–2.05)	1.98 (1.49–2.64)^***^	1.40 (1.13–1.74)^**^	1.41 (1.09–1.84)^*^
RISC total score	0.93 (0.92–0.94)^***^	0.94 (0.93–0.94)^***^	0.96 (0.95–0.97)^***^	0.97 (0.96–0.98)^***^	0.97 (0.97–0.098)^***^	0.99 (0.99–1.00)

^*^p < 0.05, ^**^p < 0.01, ^***^p < 0.001.

## 4 Discussion

From the perspective of individual-centered research, this study explores the patterns of the smartphone dependence among college freshmen by using the research method of latent profile analysis, finding out that there are four types of patterns: normal use group (11.2%), mild dependence group (37.4%), moderate dependence group (41.3%) and severe dependence group (10.1%). Among them, the severe dependence group exhibited a high proportion of negative life event exposure, a high prevalence of suicidal ideation, and extremely low psychological resilience, and the low resilience characteristics of such groups make them more prone to forming a vicious cycle during stressful events, using smartphones as the primary means of escaping reality. The four-class model is similar to some previous research results. For example, Dong et al. (2024) identified three different characteristics of smartphone dependence among college freshmen, namely, low risk (32.94%), high risk (52.75%) and high risk (14.31%). Wu et al. (2024) determined three classes of teenagers’ smartphone dependence, including “normal smartphone users” (21.50%), “smartphone addicts” (46.65%) and “high-risk smartphone users” (31.85%). These studies, along with the present research, all indicate that smartphone addiction among college students exhibits varying degrees of group classification, with risk proportions exceeding 50% and high-risk proportions surpassing 10% (Dong et al., 2024; Wu et al., 2024). In this study, a more detailed classification of the smartphone dependence among college freshmen has been made, and the results of the four classes are consistent with the number of classes divided according to the severity of traditional smartphone dependence scores. There are significant differences in the scores and trends of the four subgroups in the four dimensions of smartphone dependence: loss of control, withdrawal, escapism and inefficiency, which shows the heterogeneity of the smartphone dependence among college freshmen, which provides a unique perspective for personalized intervention on the smartphone dependence among college freshmen. Among the four subgroups of the smartphone dependence among college freshmen, three are smartphone dependence groups, accounting for nearly 90% of the total, and the number of moderate and severe smartphone dependence groups is as high as 50%, which shows that the phenomenon of smartphone dependence of college freshmen is widespread in colleges and universities in China, suggesting that it is necessary for us to carry out supportive and personalized psychological intervention for freshmen. Freshmen are in a critical period of environmental adaptation and interpersonal communication, and it is also a stage of stress susceptibility and emotional adjustment difficulties. Mental health programs tailored to stress management, digital addiction and coping strategies can effectively prevent the occurrence and development of smartphone addiction (Lee et al., 2023). Personalized cognitive behavioral therapy and psychodynamic therapy can help to find out the root causes of their addiction and the internal psychological needs behind smartphone addiction, and cultivate healthier online use habits (Lan et al., 2018). In addition, the three smartphone dependence groups all have different degrees of influence in the four dimensions of out of control, withdrawal, evasion and inefficiency, and all of them have the most outstanding average score in the dimension of evasion. It can be seen that college freshmen generally use smartphones as a medium to escape from reality, and freshmen will face problems in life and academic adaptation, while the concealment, virtuality and protection of the network serve as a safe haven for them to avoid solving practical difficulties, potentially reinforcing their dependence on smartphones.

Multinomial logistic regression analysis results show that negative life events, suicidal ideation and resilience affect the latent classes of the smartphone dependence among college freshmen. Specifically, college freshmen with suicidal ideation showed higher risk of smartphone dependence, which provides further evidence for previous studies and expands the previous research results (Huang et al., 2022; Wang et al., 2024). Wang et al. (2024) proved that college freshmen’ smartphone addiction is related to college freshmen’ suicidal ideation and attempts. Huang et al. (2022) also reported that students with suicidal ideation may have longer cell phone use time. Emerging evidence suggests that neurobiological mechanisms may underlie the association between smartphone addiction and suicidal ideation. The decrease of serotonin (5-HT) levels been implicated in both suicidal behaviors (Ambrus et al., 2016) and prefrontal cortex dysfunction associated with internet addiction (Cerniglia et al., 2020). The neurochemical pathway may partially explain the observed connection between smartphone dependence and suicide risk. In addition, the structure and function of gray matter and white matter in the prefrontal lobe of Internet addiction patients are prone to abnormal changes (Sun et al., 2023). This may be the neurobiological mechanism between cell phone addiction and suicide. In order to understand the detailed mechanism of the relationship between cell phone addiction and suicidal ideation, more cohort or biological research is needed to further confirm it. From the stress-coping theory perspective, students with suicidal ideation may use mobile phones as an escape mechanism, avoiding emotional pain through digital immersion—a maladaptive strategy in coping theory (Lazarus, 1984). This aligns with our finding that escapism is a primary dimension across all dependence groups. Self-determination theory further suggests that unmet psychological needs in students with suicidal ideation may drive excessive mobile phone use, compensating for real-world relationship deficits through virtual social connections (Deci and Ryan, 2000). Notably, many studies have confirmed the relationship between depression and suicidal ideation and the relationship is mediated by depression (Hu et al., 2022; Zhang et al., 2022). In this study, however, there is no significant influence of the history of mental illness on college freshmen’ dependence on smartphones. This paradox may stem from underreporting due to stigma or limited mental health literacy, leading students to adopt maladaptive coping strategies like smartphone overuse rather than seeking professional help. This pattern underscores the need for proactive mental health interventions in university settings, particularly mindfulness-based stress reduction and cognitive behavioral therapy (Hofmann and Gomez, 2017). This also requires universities to incorporate digital literacy education into freshman orientation courses and develop tiered intervention policies. For high-risk mobile phone addiction groups, measures such as establishing a “Digital Detox Day,” providing psychological counseling green channels and high-intensity cognitive behavioral therapy (CBT) can be implemented to specifically address the issue of college freshmen’s reality-avoidance behaviors and potential suicidal ideation risks. To address the widespread dependency phenomenon among freshmen, digital health lectures can be conducted, campus network management optimized, and usage periods for non-essential applications restricted to balance academic performance with healthy mobile phone usage. It is also found that compared to the mild dependence group, college freshmen who experience negative life events have a higher risk of entering the severe dependence group. This aligns with the hypothesis in ecosystem theory that “macro-level stressors exacerbate vulnerabilities in micro-level systems” (Bronfenbrenner, 1986). The negative life events of college freshmen usually include serious diseases, interpersonal conflicts, economic difficulties and academic setbacks. In this study, the escapism dimension of the smartphone dependence among college freshmen is relatively the highest, which shows that the excessive use of college freshmen’ smartphones has the function of avoiding the pressure of real life, forming a vicious cycle of “pressure-escape-increasing dependence” (Fu et al., 2020). Meanwhile, research shows that negative life events of college students can lead to suicidal ideation (Yang et al., 2021). This also provides us with thinking and direction for exploring the relationship and the potential shared etiology among negative life events, suicidal ideation and smartphone dependence in the future.

Psychological resilience demonstrates robust protective effects against smartphone dependence in this study, the higher the level of psychological resilience, the lower the risk of falling into the severe mobile phone dependence group. This finding extends previous research demonstrating a significant negative correlation between smartphone addiction and psychological resilience (Dong et al., 2024; Ma et al., 2022; Xie et al., 2023). Individuals with strong psychological resilience show enhanced self-management and self-restraint, and have good resistance and resilience, enabling them to use internal resources to adjust their internal needs and take active coping strategies to solve problems, thus reducing their tendency to participate in social media excessively and reducing the risk of addiction. On the contrary, college students with decreased psychological resilience show weak behavior control and insufficient self-management, which makes them more likely to overuse smartphones and lead to smartphone addiction (Tang and Lee, 2021). In fact, as a trainable skill, tiered psychological resilience training programs can be implemented, including weekly mindfulness-based cognitive therapy (MBCT) courses focused on emotion regulation and attention training, regularly organized peer support groups for social skills and stress management, effective use of digital self-monitoring tools for feedback, and cognitive behavioral workshops to help establish adaptive thinking patterns. These measures aim to enhance the adaptability of college freshmen, thereby contributing to the prevention and intervention of smartphone addiction (Denckla et al., 2020). The first is to strengthen freshmen’s metacognitive awareness training, which can help students better understand their smartphone usage patterns, enhance digital self-monitoring, and form more cautious thinking and usage habits (Smith et al., 2019). The second is to train the ability of emotional regulation, which can make college freshmen have healthier emotional management strategies, thus reducing emotional dependence on smartphones (Ching and Tak, 2017). Establishing a robust social support network for freshmen is crucial to enhancing psychological resilience. Adequate emotional and information support, as a stable resource, can help students better adapt to the challenges of college diversity, rather than just turning to smartphones as an escape mechanism (Yang et al., 2023).

### 4.1 Strengths, limitation, and prospect

From the perspective of individual-centered, this study deeply reveals the heterogeneous characteristics of the smartphone dependence among college freshmen and the specific factors affecting it, which provides a brand-new perspective for preventing and intervening the smartphone dependence among college freshmen and helps to formulate more targeted prevention and treatment strategies. The study also emphasizes the influence of suicidal ideation, negative life events and psychological resilience on smartphone dependence, which provides a screening and key attention direction for mental health education and prevention of smartphone dependence and has important practical guiding significance. There are still some shortcomings in this study. Firstly, this study uses cross-sectional data, which cannot infer the quantitative relationship in time, which limits the ability of causal inference. Secondly, this findings are based on college freshmen from a single university in Eastern China, where smartphone addiction patterns may be influenced by localized cultural and educational norms. Thirdly, these data are self-reported and may be influenced by subjective bias. Finally, this study failed to encompass all potential predictive factors. Therefore, future research should recruit participants from multiple regions and cultural contexts to test the generalizability of these latent classes, and integrate various methods, such as observation research, control experiments and digital tracking of actual equipment usage to reduce self-report biases, future research should also employ longitudinal designs to track the developmental trajectories of smartphone addiction and explore the temporal relationships between predictive factors including personality traits or academic stress and outcomes.

## 5 Conclusion

This study found that the smartphone dependence among college freshmen is heterogeneous, and could be categorized into four classes: normal use group, mild dependence group, moderate dependence group and severe dependence group. Negative life events, suicidal ideation and resilience affect the latent classes of the smartphone dependence among college freshmen. Our findings inform campus mental health strategies, urging integration of smartphone dependence screening into freshman mental health screenings, consciously organizing group psychological resilience training program to help college freshmen effectively cope with and manage stress and improve their self-control ability in the rational use of smartphones.

## Data Availability

The raw data supporting the conclusions of this article will be made available by the authors, without undue reservation.
