# Supplementary material for: Patterns of smartphone dependence and predictive factors in Chinese college freshmen: a latent profile analysis
Source: Front Psychol. 2025 Jun 18;16:1592875. doi: 10.3389/fpsyg.2025.1592875 (PMC12213825; doi:10.3389/fpsyg.2025.1592875)
Supplement: Supplementary file 1 [file Table_1.docx]

**Table S1.** Socio-demographic Characteristics of Participants

| Variable | Category | n | Percentage (%) | Variable | Category | n | Percentage (%) |
| --- | --- | --- | --- | --- | --- | --- | --- |
| Gender | Female | 2349 | 48.3 | Peer Relationship | Harmonious | 4534 | 93.2 |
|  | Male | 2514 | 51.7 |  | Discordant | 329 | 6.8 |
| Origin | Urban | 1641 | 33.7 | Negative Life Events | Yes | 439 | 9 |
|  | Rural | 3222 | 66.3 |  | No | 4424 | 91 |
| Family Ranking | Only Child | 1770 | 36.4 | Left-behind Experience | Yes | 799 | 16.4 |
|  | Non-only Child | 3093 | 63.6 |  | No | 4064 | 83.6 |
| Family Structure | Complete | 4356 | 89.6 | Mental Illness | Yes | 93 | 1.9 |
|  | Single Parent | 507 | 10.4 |  | No | 4770 | 98.1 |
| Family Economic Status | Well-off | 1036 | 21.3 | Suicidal Ideation | Yes | 596 | 12.3 |
|  | Common | 3152 | 64.8 |  | No | 4267 | 87.7 |
|  | Poor | 675 | 13.9 |  |  |  |  |
